# Can Clinical Trials Requiring Frequent Participant Contact Be Conducted Over the Internet? Results From an Online Randomized Controlled Trial Evaluating a Topical Ointment for Herpes Labialis

**DOI:** 10.2196/jmir.6.1.e6

**Published:** 2004-02-17

**Authors:** Margaret Formica, Karim Kabbara, Rachael Clark, Tim McAlindon

**Affiliations:** ^1^Divisions of Rheumatology & Clinical Care ResearchTufts-New England Medical CenterBoston MA 02111USA; ^2^Office of Information TechnologyBoston University School of MedicineBoston MA 02118USA; ^3^Department of DermatologyBrigham and Women's HospitalBoston MA 02115USA

**Keywords:** Internet, randomized controlled trial, clinical trial, herpes labialis, dioctyl sodium sulfosuccinate

## Abstract

**Background:**

The Internet has tremendous appeal for conducting randomized clinical trials and may be especially applicable to trials requiring frequent participant contact. Trials of cold sore remedies, for example, often require daily clinic visits during outbreaks, imposing substantial burden on participants. An Internet-based randomized clinical trial design may reduce this burden, permitting frequent symptom reports with considerably less effort.

**Objective:**

To evaluate the feasibility of a Web-based randomized clinical trial requiring frequent participant interaction, using a 6-month, double-blind, randomized, placebo-controlled pilot trial of a topical ointment containing dioctyl sodium sulfosuccinate (DSS) (Zilex; Meditech Pharmaceuticals, Inc, Scottsdale, Arizona, USA) intended for treatment of recurrent herpes labialis. A secondary objective was to obtain preliminary data on effectiveness outcomes, to assist in planning a fully-powered trial of DSS.

**Methods:**

Adults with physician-confirmed herpes labialis were recruited to apply to the trial. Eligible applicants were randomized to DSS or placebo, mailed to them upon enrolment with instructions to apply topically every 2 hours for the duration of every cold sore outbreak. Participants were instructed to complete online questionnaires at 2-week intervals and, at the initiation of a cold sore, daily "outbreak questionnaires" until outbreak termination. Feasibility outcome measures included trial participant characteristics, frequency of cold sores, participant retention and adherence (to study medication), and data completeness. Treatment effectiveness outcome measures included outbreak duration, days to crust formation, and pain.

**Results:**

Of the 292 individuals applying, 182 screened eligible; 32 participants with confirmed herpes labialis enrolled in the trial. 16 were randomized into the verum group and 16 into the placebo group. 29 (91%) participants completed the trial. During the trial, 34 outbreaks were reported among 23 (72%) participants, resulting in a cold sore incidence rate of 19.8 per 100 person-months of observation. Online data were available for 32 outbreaks; the absence of a resolution date made it impossible to accurately calculate the duration of 12 (38%) outbreaks. Although the DSS treatment group had a shorter mean outbreak duration (6.6 vs 7.7 days, *P*= .2) and fewer mean days to crust formation (3.5 vs 4.9, *P*= .1), these differences did not reach statistical significance. The DSS group has statistically significant lower mean pain scores (3.1 vs 7.6, *P*= .04), but participants in this group also consumed more acetaminophen tablets than the placebo group (1.1 versus 0.5, *P*=.55). Adherence to medication was similar in both groups: 7 (50%) of the verum group reported using the cream as directed compared to 6 (46.2%) in the placebo group; ( *P*= .8).

**Conclusions:**

We efficiently recruited participants and achieved high overall retention rates. However, participant adherence to the daily outbreak visit schedules was low and only 7 (50%) participants used the cream as directed. These limitations could be addressed in future Internet-based studies by using Personal Digital Assistants (PDAs), using reminder devices, and providing incentives. By enhancing participant adherence, clinical trials requiring frequent participant contact may be feasible over the Internet.

## Introduction

Due to its access to vast segments of the population and its technological capabilities, the Internet has tremendous appeal as a vehicle for conducting randomized controlled clinical trials (RCTs) [1- 4]. In previous work we found it highly feasible to rapidly recruit individuals over the Internet into an online randomized controlled clinical trial of glucosamine for knee osteoarthritis [5]. The Internet-based approach may be especially applicable to randomized controlled clinical trial designs that require frequent participant contact. Trials of cold sore remedies, for example, often require daily clinic visits during outbreaks, imposing substantial burden on participants [6]. While clinic visits may represent the ideal patient evaluation, these frequent visits may hinder participant recruitment and retention. An Internet-based randomized controlled clinical trial design could theoretically reduce this burden and permit frequent symptom reports with considerably less effort.

The objectives of our study were to evaluate the feasibility of an Internet-based approach for testing a topical ointment containing dioctyl sodium sulfosuccinate (DSS) (Zilex; Meditech Pharmaceuticals, Inc, Scottsdale, Arizona, USA) for herpes labialis (oral herpes simplex, cold sores), and to collect preliminary outcome data for this potential cold sore remedy. DSS has been shown to have in vitro efficacy against herpes simplex virus type 1 through disruptive effects on the viral capsule [7]. In addition, Zilex contains benzocaine, a well-known anesthetic that has been shown to be effective in pain reduction [8,9].

## Methods

### Web Site Development

The study Web site was constructed on an independent server within the Boston University School of Medicine domain. The Web pages were written in Hypertext Markup Language (HTML), Active Server Pages (ASP), JavaScript, and Component Object Modeling interfaced with a Structured Query Language (SQL) Server database. Microsoft Access [10] software was used to query the database and to create reports. The security of our site was protected by a firewall and 128-bit secure socket layering encryption. In addition, we operated data-security protocols that included automated error checking, limited password-protected access to the data interface and database, permission levels, username-linked logging of all changes made in the database, participant tracking by ID number, encryption of randomization codes, and screen time-outs.

The public area of the Web site described the study and solicited participants, with hypertext links to a consent form and an eligibility-screening page. The password-protected private area of the Web site included pages where participants could view utilities and access the study questionnaires. The Web site included a batching utility that (1) sent e-mails to participants reminding them of their scheduled online "visits" and (2) included code that presented the appropriate questionnaires to each participant when logging into the Web site at the time of these visits.

### Design of the Randomized Controlled Clinical Trial

This was a 6-month randomized, placebo-controlled trial of a topical ointment in the treatment of herpes labialis. The objective was to enroll 30 participants with a history of herpes labialis, randomize them to verum (Zilex) or placebo, and follow them for cold sore outbreaks. Because of the 6-month follow-up period, participants had the opportunity to report several cold sore outbreaks and the key emphasis of the trial was on the "outbreak." The primary outcome measure was duration of outbreak, which was ascertained through daily outbreak questionnaires. This study was approved by the Institutional Review Board at Boston University School of Medicine.

### Recruitment and Eligibility

Adults with a history of herpes labialis were recruited through newspaper and e-zine advertisements directing them to the trial Web site, which described the study and contained an eligibility questionnaire. Eligible applicants had to be at least 18 years of age, take no immunosuppressives, report at least 4 cold sore outbreaks per year for more than 1 year, report a last-outbreak occurrence ≤ 6 months ago, and report at least moderately-severe external cold sores. Applicants who screened eligible were asked to print, sign, and mail an informed consent form including a medical-records release. When medical records could not be obtained, the participant was contacted by a physician (TM) to confirm the diagnosis of herpes labialis. The identity of all participants was authenticated by receipt of a signed consent form. In addition, authentication of participant's identities was confirmed by receipt of medical records, or by telephone.

### Randomization, Medication Delivery, and Medication Application

Eligible authenticated applicants who completed a 2-week run-in phase were randomized in a double-blind manner to either verum or placebo. Staff uninvolved with other aspects of the trial maintained the computer-generated randomization list and labeled the tubes of study medication. The tubes, distinguishable only by a code number on the label, were then provided to study staff. Tubes of study medication, along with an instruction guide, were sent to participants by 2-day mail. Participants were sent an e-mail notifying them that the study medication was en route and should arrive within 5 days. Participants were asked to notify us via the study Web site when they received the study medication. Participants were instructed to apply the study medication at the first sensation of a cold sore outbreak and continue to apply the medication every 2 hours until the cold sore healed completely.

### Questionnaires

Participants were asked to complete scheduled online questionnaires at 2-week intervals; these questionnaires included information on the participant's cold sore history and remaining study medication.

**Figure 1:**
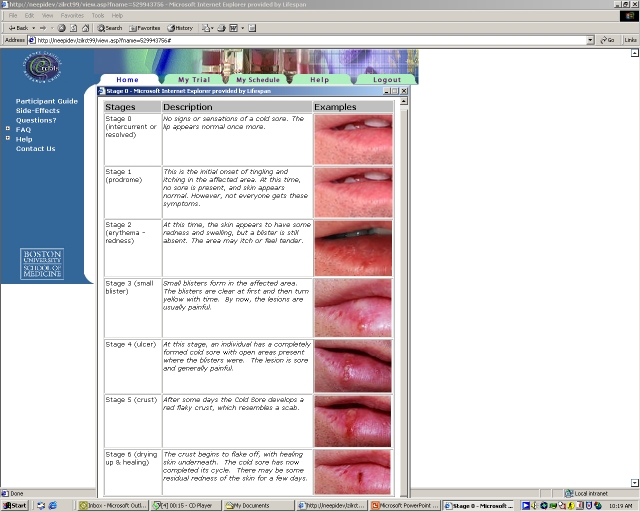
Standard Pictorial Illustration of Cold Sore Stages as Part of the Online Outbreak Questionnaire

**Figure 2:**
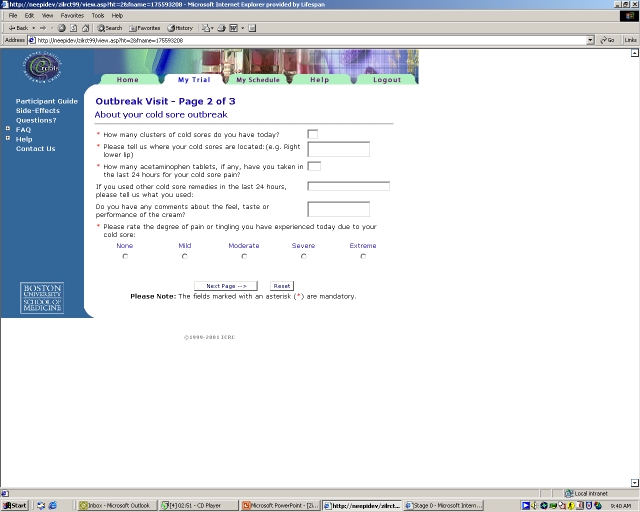
Part of the Outbreak Questionnaire (see also Multimedia Appendix)

At the initiation of an outbreak, participants were asked to complete daily online outbreak questionnaires, which were date stamped and included a standard pictorial illustration of cold sore evolution (stages 1-6, and 0 indicating a completely-healed cold sore; Figure 1), as well as questions on the number of clusters present, the location of the cold sore, use of pain medication or other cold sore medications, and the degree of pain associated with the cold sore (Likert scale 0-4) (see Figure 2 and Multimedia Appendix). E-mail reminders were sent to participants if they did not complete a daily outbreak questionnaire during the course of an outbreak. A supplemental questionnaire was e-mailed to participants at the completion of a cold sore outbreak; this questionnaire asked about the length of the outbreak, how long ago the outbreak occurred, adherence to study medication, and satisfaction with the study medication.

### Outcome Measures

Outcome measures pertaining to the evaluation of the feasibility of conducting the trial online are descriptive in nature and include characteristics of the trial participants, frequency of cold sore outbreaks, participant retention and adherence to the study medication, and completeness of data.

Treatment-effectiveness measures included total duration of outbreak, days to crust formation, and pain. Total duration of outbreak was defined by subtracting the date of the initial online outbreak questionnaire from the date of the outbreak questionnaire that indicated the cold sore had healed completely. Days to crust formation was defined by subtracting the date of the initial online outbreak questionnaire from the date of the online questionnaire in which the participant first reported that the cold sore had reached at least stage 5. Pain was defined as the sum of the daily pain scores for the duration of the outbreak. Additional outcome measures included sum of the daily scores for cold sore stage, sum of the number of clusters reported daily for the duration of the outbreak, and the sum of acetaminophen tablets taken over the duration of the outbreak.

### Adverse Events

Participants were asked to report any adverse events via a form on the Web site, e-mail to the study staff, or a toll-free telephone line. The daily online outbreak visit questionnaire also allowed participants to report comments on the feel, taste, or performance of the study cream. In addition, participants were asked in the supplemental e-mailed questionnaire if they had any problems with the study cream.

### Statistical Analysis

We used an intention-to-treat analytic approach. In cases where the total duration of an outbreak could not be calculated because the endpoint of the outbreak was unclear or the participant informed us of the outbreak only after it occurred (N = 2), we imputed a duration based on the mean duration of all of the other outbreaks. This method was used for the days to crust formation, sum of the daily pain scores, sum of the daily stage scores, sum of the number of clusters reported daily, and sum of acetaminophen tablets taken over the duration of the outbreak. Additional analyses were conducted excluding outbreaks for which imputation was necessary.

Baseline characteristics of the treatment groups were compared using Fisher exact tests and *t* tests. Differences between the treatment groups were evaluated using Wilcoxon rank sum tests for continuous outcome measures and Fisher exact tests and chi-square tests for categorical outcome measures. Generalized linear models were used to test for differences after adjusting for gender and age.

### Results

**Figure 3:**
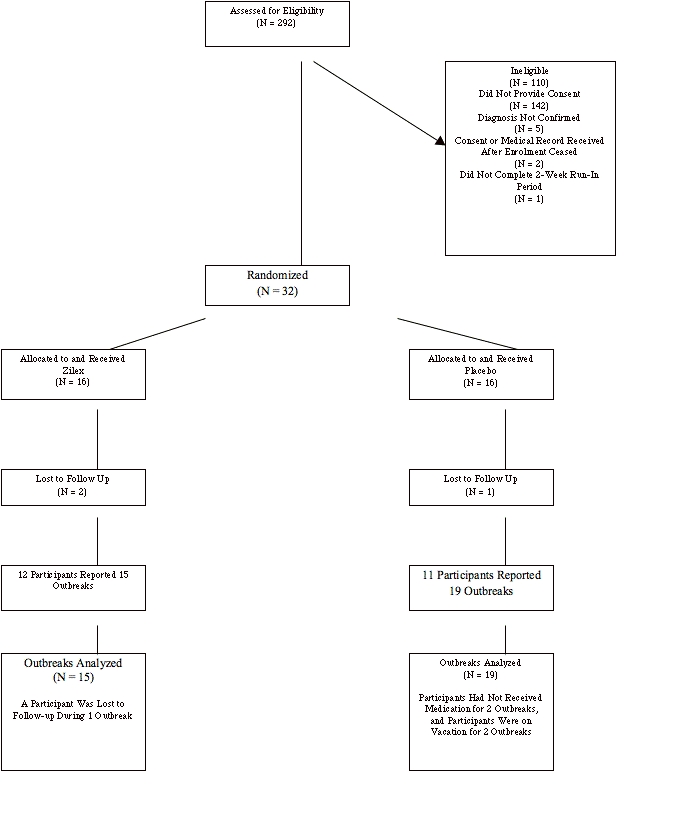
CONSORT flow diagram

Figure 3 shows the CONSORT flow diagram [11] of the trial from application to completion. Of the 292 individuals who applied to the trial, 182 screened eligible, and 40 mailed signed consent forms. Ultimately, 32 participants with confirmed herpes labialis completed the 2-week run-in phase and enrolled in the trial, verum (N = 16) or placebo (N = 16).

The mean age of participants was 43 (range, 20-72). 23 (72%) were female and all participants were Caucasian. Baseline characteristics of the participants by randomized group are displayed in Table 1. The groups were similar with respect to geographic region, but differed with respect to age and gender, although these differences did not reach statistical significance on a 5% level.

Three participants were lost to follow-up, resulting in 29 (91%) who completed the trial. During the course of the trial, 34 outbreaks were reported among 23 (72%) participants. After accounting for losses to follow-up, the cold sore incidence rate was 19.8 per 100 person-months of observation. Two outbreaks occurred while participants were on vacation, leaving 32 outbreaks for which online data were available.

**Figure 4:**
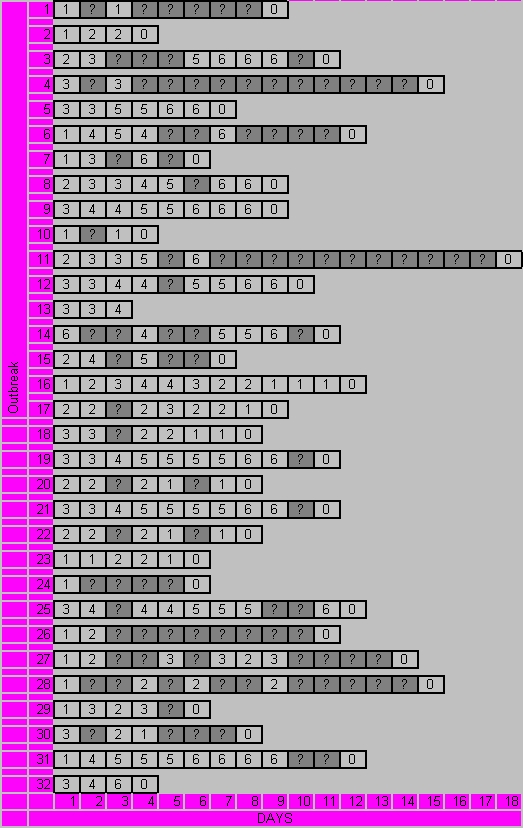
Duration and daily stage information for 32 cold sore outbreaks for which online data was available. Outbreaks 1-15 were treated with DSS, outbreaks 16-32 were treated with placebo. ? = missing data

Figure 4 illustrates the completeness of data, as well as self-reported stage for the 32 cold sore outbreaks for which we had online data. Due to missing data at the end of the outbreak, total duration of outbreak could not be calculated for 12 (38%) and days to crust formation could not be calculated for 10 (31%) of the outbreaks. The stages for 14 (44%) outbreaks followed a normal cold sore evolution.

Results adjusted for gender and age did not differ substantially from unadjusted results, therefore, only the former are presented. The verum group had a shorter mean duration of outbreak, fewer mean days to crust formation, and lower mean sum of clusters than the placebo group, but none of these differences reached statistical significance. The verum group had a statistically significant ( *P*=.04) lower mean sum of daily pain scores than the placebo group (Table 2). The mean sum of acetaminophen tablets taken over the course of the outbreak was slightly higher in the verum group compared to the placebo group. Analyses that excluded outbreaks for which imputation was necessary had similar results, but the magnitude of difference between the treatment groups was greater.

**Table 1:** Baseline characteristics of trial participants

	Participants Receiving DSS (N = 16) N (%)	Participants Receiving Placebo (N = 16) N (%)	P*
Female	9 (56.3)	14 (87.5)	.11
Region of the United States			
Northeast	7 (43.8)	6 (37.5)	.42
Southeast	2 (12.5)	5 (31.3)	
Midwest	5 (31.3)	2 (12.5)	
West	2 (12.5)	3 (18.8)	
Mean age (SD)	39.8 (10.5)	46.2 (13.4)	.14
Median age (IQ range)	44.5 (15)	51.5 (14)	

*P* values are based on 2-tailed Fisher exact tests for categorical outcome measures and a 2-tailed, unpaired *t* test with 30 degrees of freedom for mean age.

Adherence to the study medication was similar in both groups, 7 (50%) of the DSS group reported using the cream at least every 2 hours compared to 6 (46.2%) in the placebo group; ( *P*= .84). Most participants reported using the cream at least every 4 hours (78%) and all participants reported using the cream at least once per day during an outbreak. For 2 outbreaks, participants had not yet received their study medication in the mail. Both groups were similar with respect to belief that they were taking the active cream, that the cream made their cold sores much better, helped abort their outbreaks, and worked as well or better than the creams used in the past (Table 3).

**Table 2:** Means of duration of outbreak, days to crust formation, sum of pain scores, sum of stage scores, sum of clusters, and sum of acetaminophen use by treatment group; adjusted for gender and age

	Outbreaks (N = 15) for Participants Receiving DSS	Outbreaks (N = 19) for Participants Receiving Placebo	P
Total duration of outbreak (days)	6.6	7.7	.24
Days to crust formation	3.5	4.9	.10
Sum of daily pain scores	3.1	7.6	.04
Sum of daily stage scores	17.5	17.9	.95
Sum of clusters	4.7	6.8	.24
Sum of acetaminophen tablets	1.1	0.5	.55
After exclusion of outbreaks requiring imputation			
Total duration of outbreak (days)*	6.3	8.2	.21
Days to crust formation†	3.5	5.0	.14

^*^ Six and 8 outbreaks were excluded from the verum and placebo groups, respectively.

^†^ Five and 7 outbreaks were excluded from the verum and placebo groups, respectively.

**Table 3:** Frequencies, percentages, and 2-tailed Fisher exact tests of categorical outcome measures

	DSS (N = 14) N (%)	Placebo (N = 13) N (%)	P
Reported use of cream as directed	7 (50.0)	6 (46.2)	.84*
Reported cream made cold sores "much better"	8 (57.1)	9 (69.2)	.69
Reported cream helped abort outbreak	9 (64.3)	10 (76.9)	.68
Reported belief that they are taking active cream	13 (92.9)	10 (76.9)	.33
Reported cream worked "as well or better than creams used in past"	10 (76.9)†	12 (92.3)	.59

^*^ Chi-square test (χ ^2^_1_= 0.04).

^†^ Information was missing for 1 outbreak.

No adverse events were reported during the course of the trial via the adverse-event reporting form on the Web site, e-mail, or phone. However, several participants reported in questionnaires that:

- the cream tingled/burned on application: DSS (n = 1), placebo (n = 3)
- was numbing: DSS (n = 5), placebo (n = 1)
- was gritty: DSS (n = 1), placebo (n = 1)
- dried the lips: DSS (n = 1), placebo (n = 0)
- tasted bad: DSS (n = 1), placebo (n = 2)
- did not work: DSS (n = 0), placebo (n = 2)
- slid off the lip: DSS (n = 1), placebo (n = 0)
- "made the blisters spread more": DSS (n = 1), placebo (n = 0).

### Discussion

The primary goal of this study was to evaluate the feasibility of conducting a clinical trial on the Internet that focused on outbreaks and required frequent participant contact. Certain aspects of this trial design proved to be robust. Participant recruitment was relatively easy; the interactive nature of the Web site with an eligibility questionnaire reduced the burden on the investigators of screening applicants, and we were able to exceed our recruitment goal in a short period of time (6 months). Overall participant retention in the trial was high; 29 (91%) of participants completed the trial. In addition, the reduction in pain seen in the verum group compared to the placebo group lends support to the internal validity of this approach, as Zilexcontains benzocaine, an anesthetic shown to be effective in pain reduction [8,9].

While we found recruitment to be unproblematic and overall participant retention high, there were several aspects of this clinical trial design that need refinement. While it is unclear whether this experience differs from traditional clinical trials [6], there were a number of outbreaks for which it was difficult to assign a total duration due to missing data. Many of the missed visits occurred during weekends and holidays. This pattern indicates the possibility that many participants were accessing the trial Web site from work, and may not have had access to the Internet at home. Future trials of this nature may benefit by requiring participants to have Internet access at home. In addition, our trial participants did not receive any compensation. It is conceivable that some form of compensation might improve compliance in this respect.

Another concern with the results of the trial was incomplete adherence to the study medication. It is unclear whether participants in a traditional clinic setting would have been more likely to use the study medication as directed. Poor adherence to study medication in clinical trials can have detrimental effects on the evaluation of safety and efficacy. On the other hand, the adherence rate found in this trial may be reflective of adherence to the medication in the general population and could, therefore, provide a valid evaluation of effectiveness.

Limited generalizability may be a potential limitation of an Internet-based trial design. Our participants were Internet users and might not be representative of all individuals with cold sores. While this could theoretically influence the generalizability of our findings, it should not impair the validity. Of course, hospital-based or clinic-based trials face similar problems in that their participants are frequently highly selected.

One of the primary benefits of an Internet-based design for a clinical trial requiring frequent participant contact is the reduced burden on participants. An alternative approach would be to augment this system using electronic data capture through wireless personal digital assistants (PDAs) [12].

As a feasibility study, this endeavor has been highly informative. In particular, it appears that even with the reduced participant burden inherent in the Internet-based approach, the goals of daily online visits and adherence to an every-2-hours dosing schedule pose significant challenges. Based on our experiences, we make the following recommendations for future such trials: (1) mail a supply of (masked) active and placebo to each participant at enrolment, (2) randomize outbreaks rather than participants, (3) use the Internet as a portal enhanced by wireless PDAs for frequent data capture, (4) program the PDAs to perform a reminder function for medication adherence, and (5) consider providing an incentive.

## Multimedia Appendix

Screenshots of the web-based interfaces for trial participants:

## Abbreviations

DSS: Dioctyl Sodium Sulfosuccinate
PDA: Personal Digital Assistant
