# Supplementary figures and images for: Can Clinical Trials Requiring Frequent Participant Contact Be Conducted Over the Internet? Results From an Online Randomized Controlled Trial Evaluating a Topical Ointment for Herpes Labialis
Source: J Med Internet Res. 2004 Feb 17;6(1):e6. doi: 10.2196/jmir.6.1.e6 (PMC1550589; doi:10.2196/jmir.6.1.e6)

## Slide 1
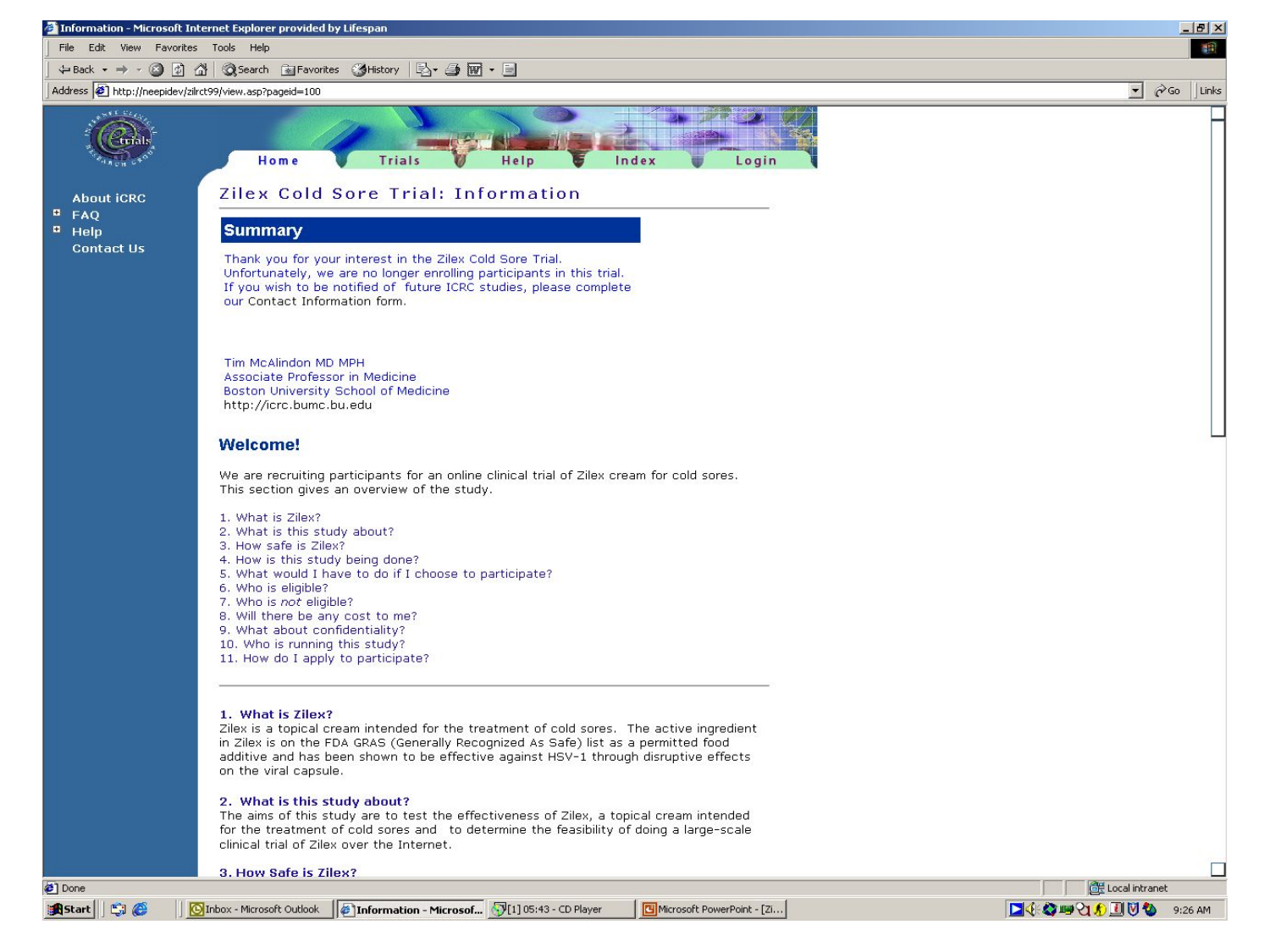

## Slide 2
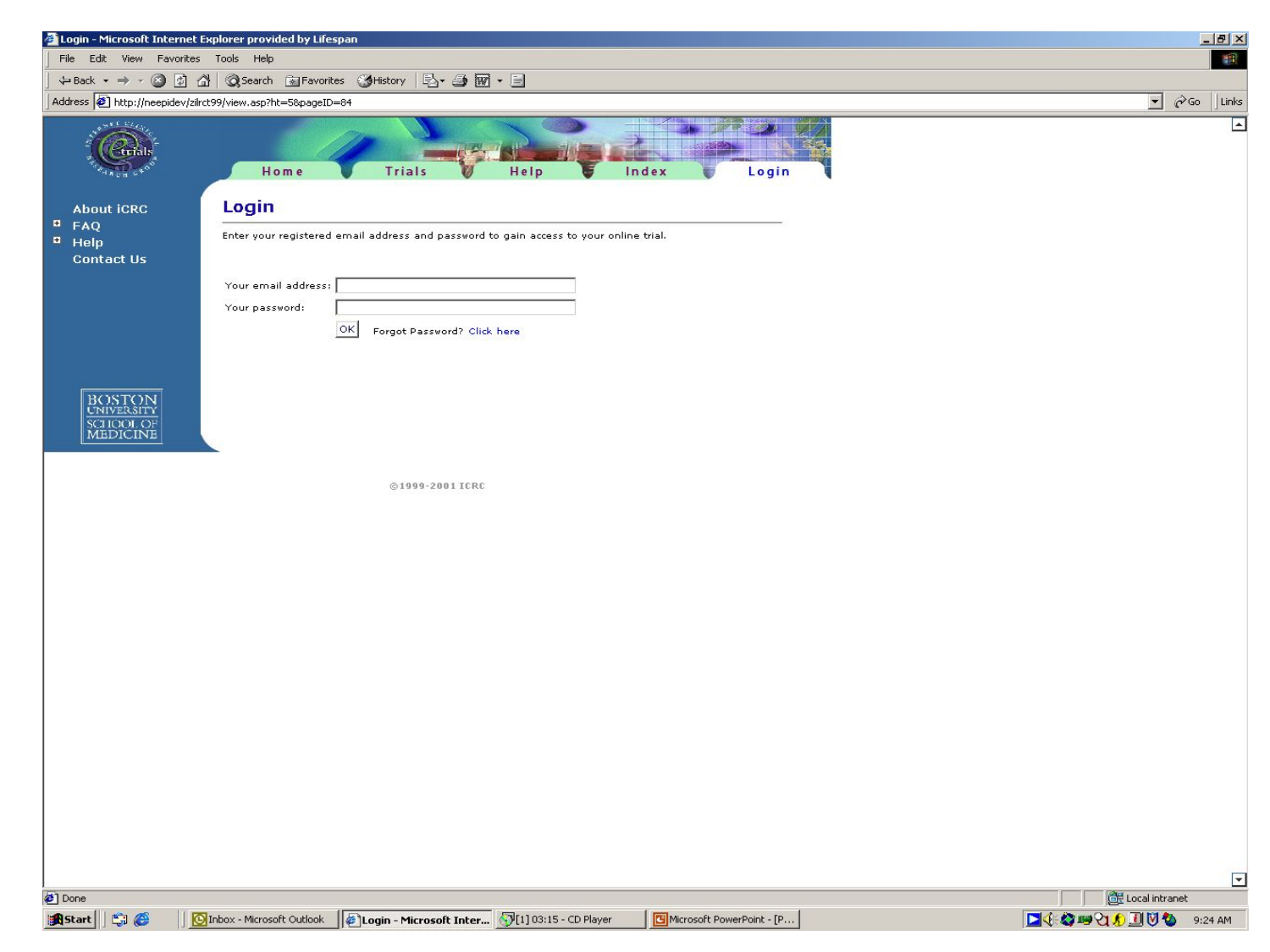

## Slide 3
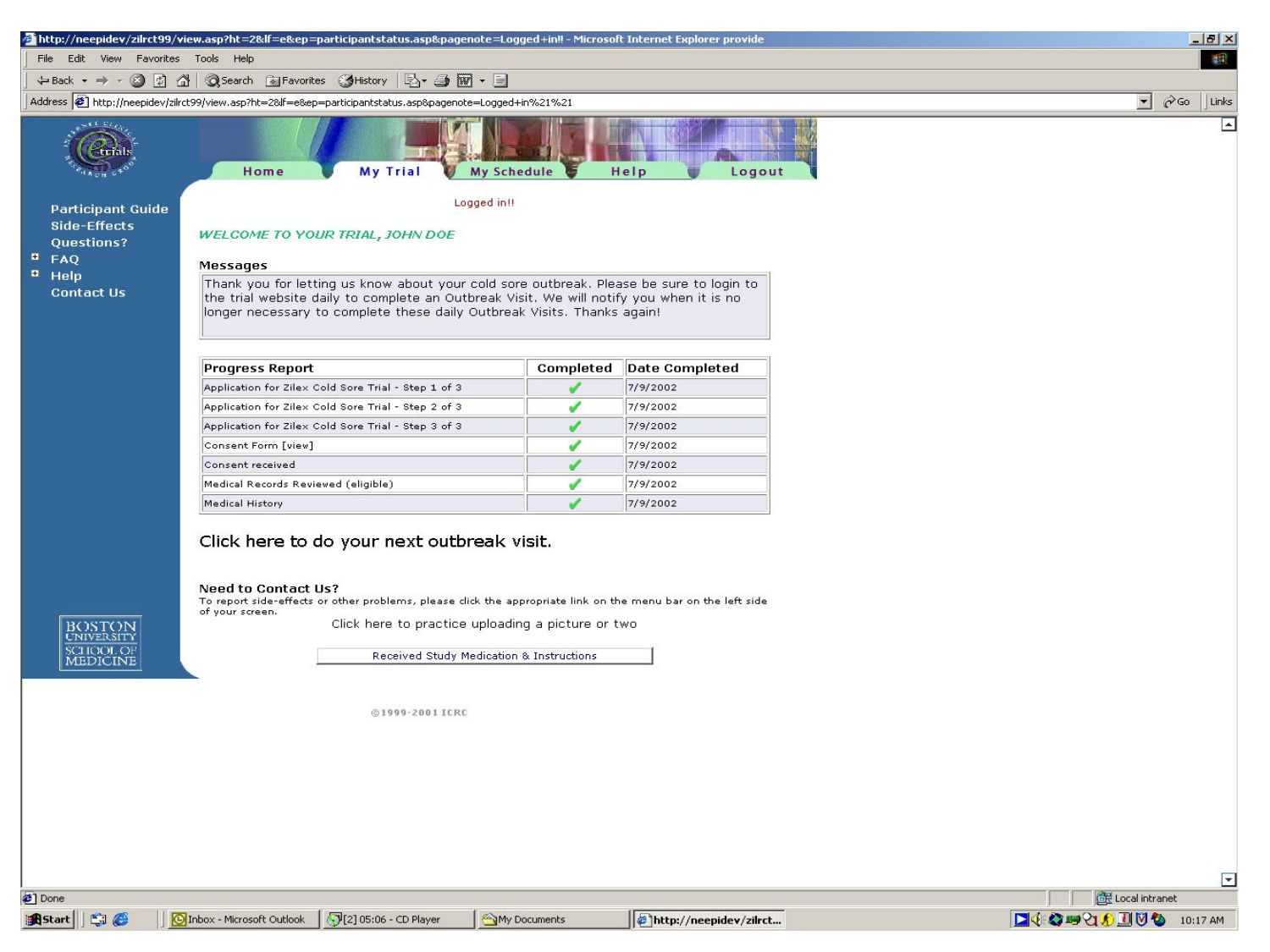

## Slide 4
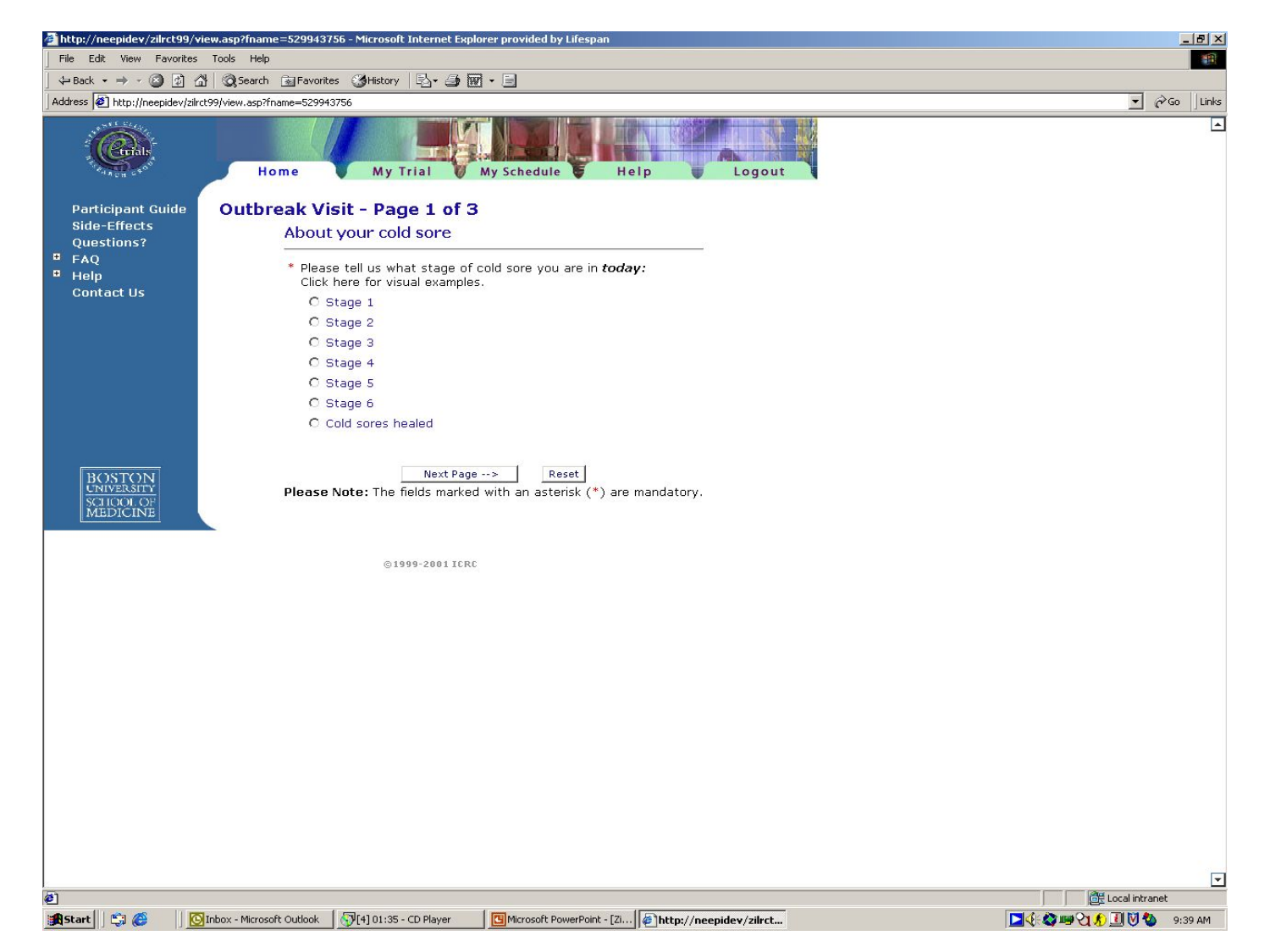

## Slide 5
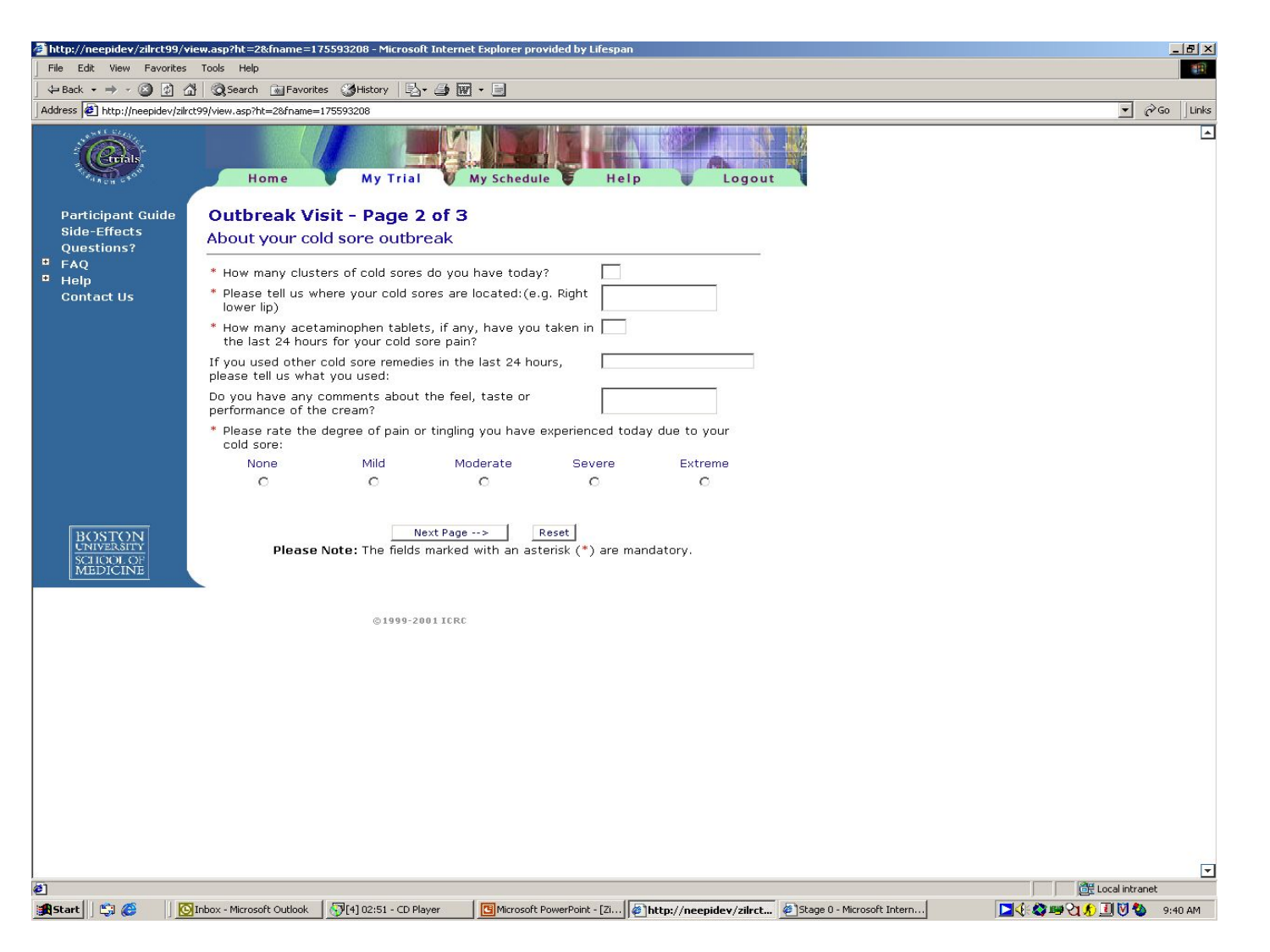

## Slide 6
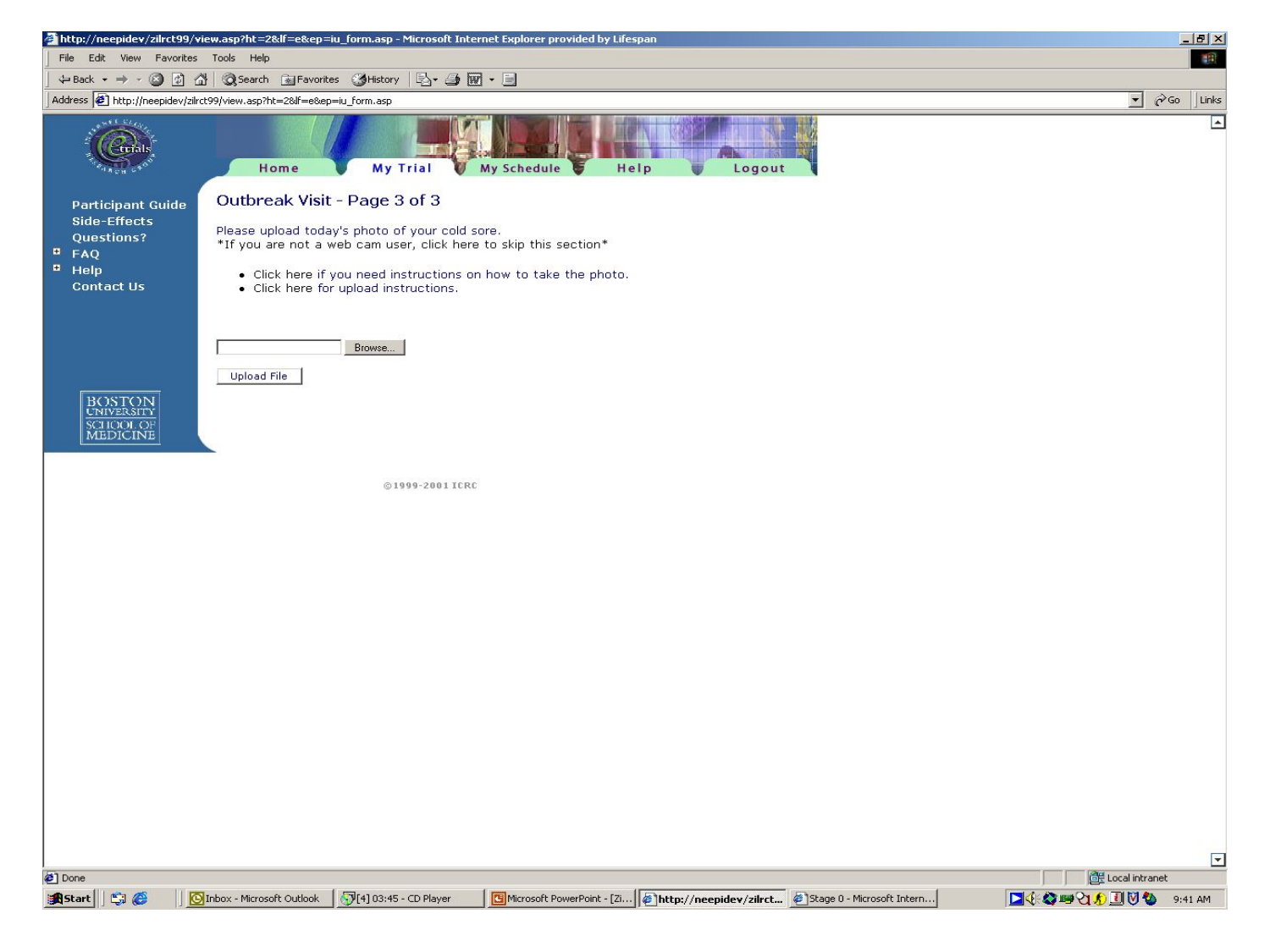

## Slide 7
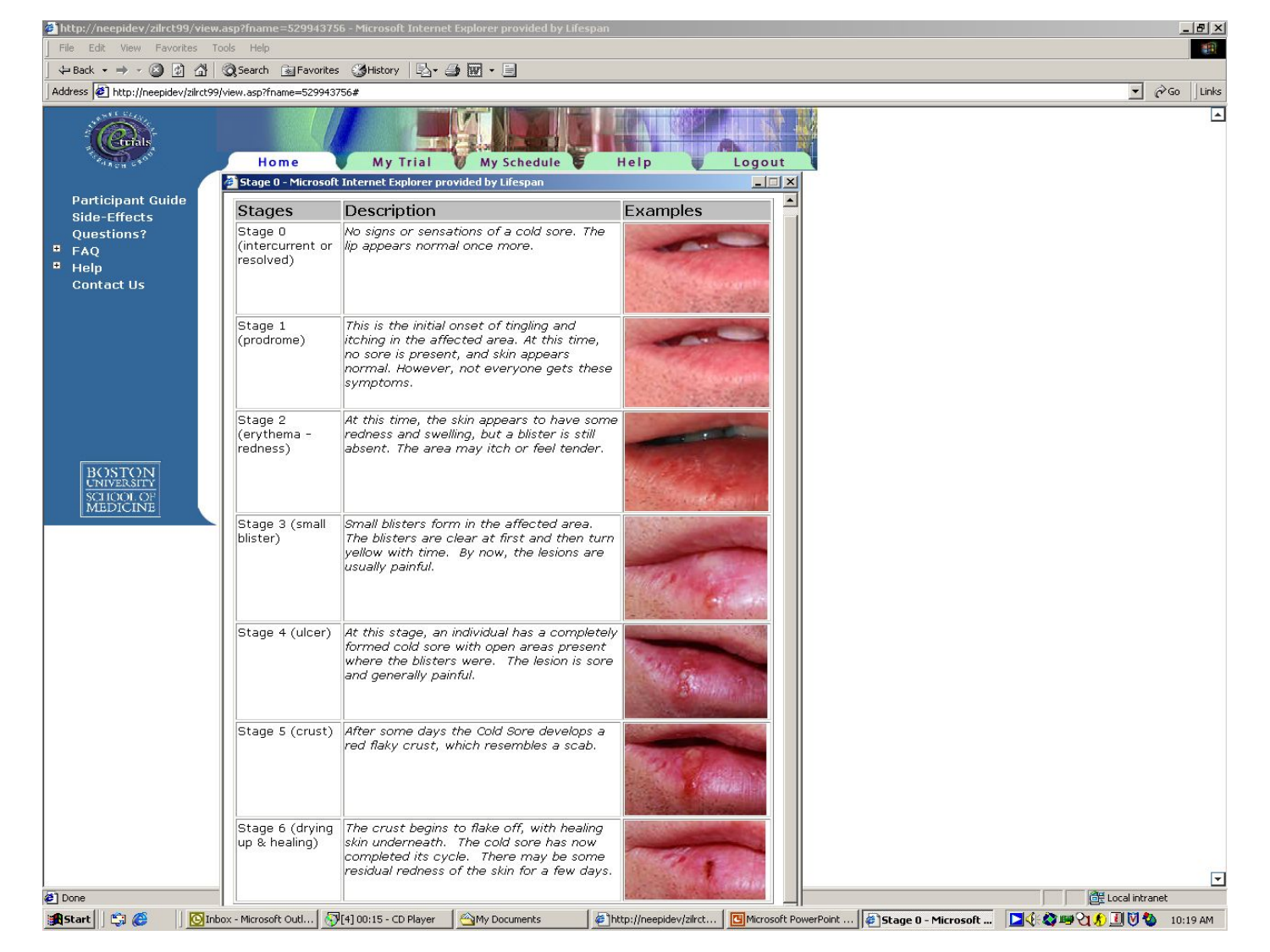

## Slide 8
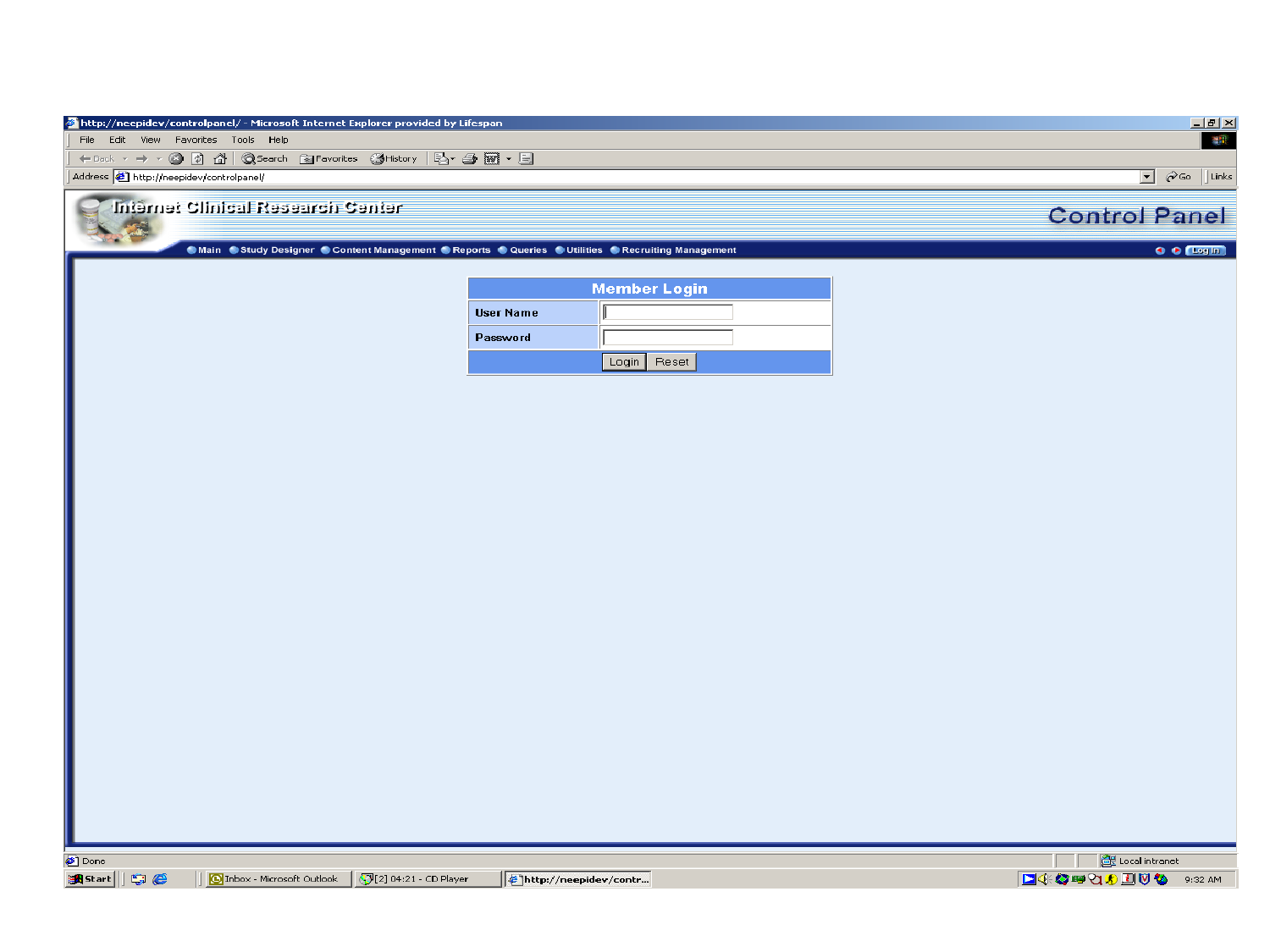

## Slide 9
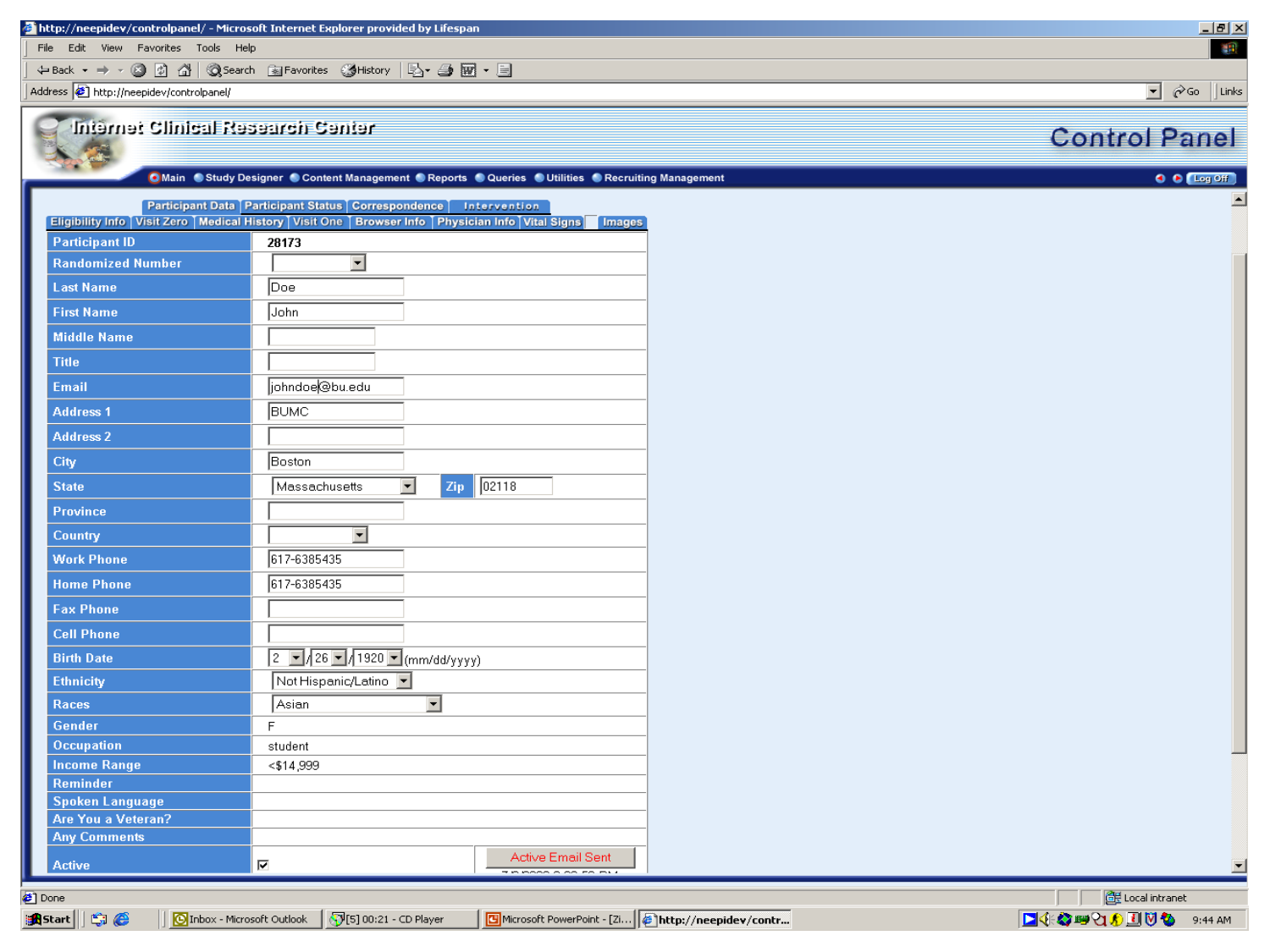

Supplement: Supplementary file 1 [file jmir_v6i1e6_app1.ppt]
